# Supplementary material for: Persistence of Chronic Lymphocytic Leukemia Stem-like Populations under Simultaneous In Vitro Treatment with Curcumin, Fludarabine, and Ibrutinib: Implications for Therapy Resistance
Source: Int J Mol Sci. 2024 Feb 7;25(4):1994. doi: 10.3390/ijms25041994 (PMC10888954; doi:10.3390/ijms25041994)
Supplement: Supplementary file 1 [file ijms-25-01994-s001.zip › ijms-2755756-supplementary.pdf]

Supplemental Materials

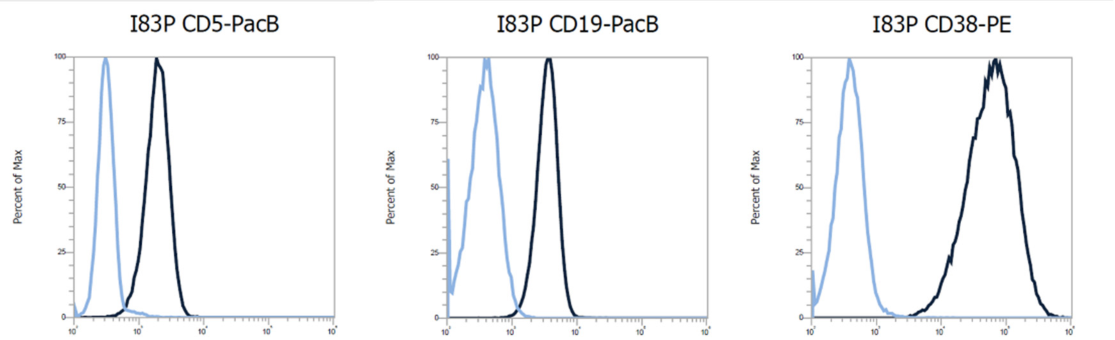

Figure S1. Immunophenotyping of I83 cells. Immunophenotyping showed that both populations, diploid and tetraploid, were positive for CD5, CD19, and CD38.

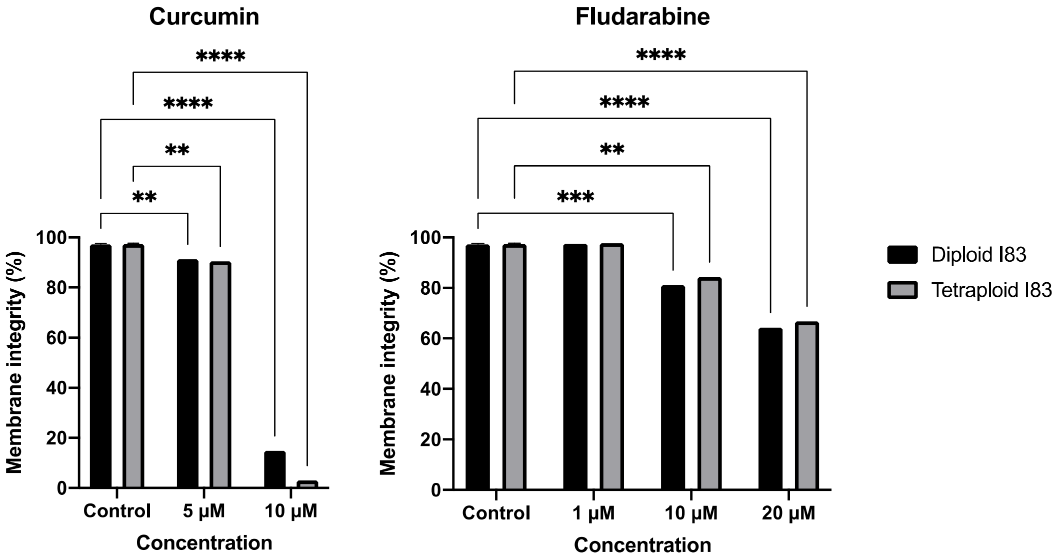

Figure S2. Comparative analysis of sensitivity of diploid and tetraploid I83 to curcumin and fludarabine. Diploid and tetraploid I83 cells were incubated for 72 h at 37°C and 5% CO_2 with different curcumin (5 μM and 10 μM) and fludarabine (1 μM, 10 μM and 20 μM) concentrations. Curcumin and fludarabine produced similar effects on cell membrane integrity of diploid and tetraploid I83. Comparing control cells with cells treated with various concentrations of curcumin and fludarabine, significant differences in membrane integrity were observed in both diploid and tetraploid I83 cell populations. ** $P < 0.05$; ** $P < 0.01$; *** $P < 0.001$; **** $P < 0.0001$.*

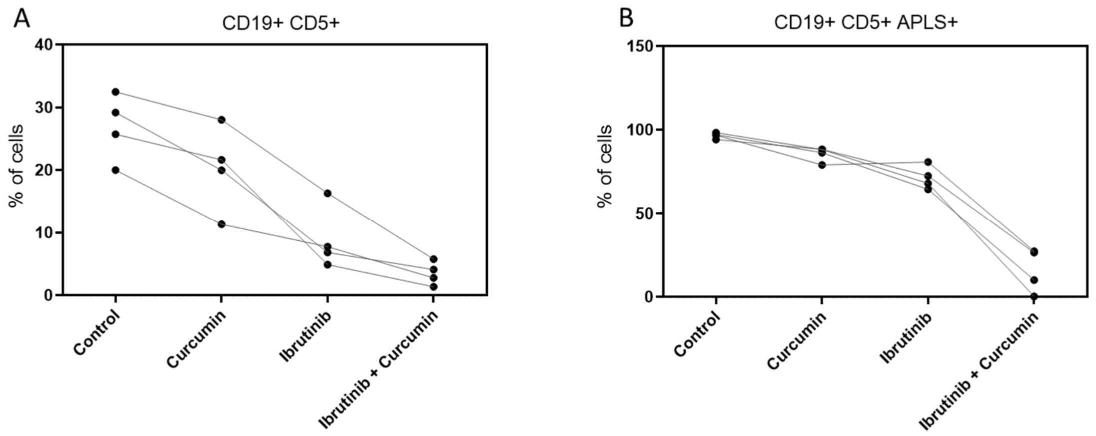

Figure S3. Curcumin synergistic effect combined with ibrutinib in P4 follow-ups. (a) Progressive reduction of CD19 CD5 cells was observed at every follow-up. Response to curcumin and ibrutinib of the cells was maintained, achieving in all cases similar effects. (b) No changes were observed for CD19 CD5 APLS cells along the treatment. For all follow-ups, response rates were consistently high near 100%, and the response to ibrutinib and curcumin was remarkably similar. However, no significant effects were observed due to the reduced sample size.

Table S1. Curcumin and drug effects on primary cell cultures.

Patient	Treatment	% of cells			Incubation time
		PI -	CD5 + CD19 +	CD5 + CD19 + APLS +	
		**FLUDARABINE**			
P5	Control	13,081	30,703	74,046	72h
	Curcumin	10,87	33,003	70,155	
	Drug	5,497	60,788	42,473	
	Combination	4,393	43,076	39,666	
P6	Control	22,657	67,293	43,267	72h
	Curcumin	21,542	64,51	43,142	
	Drug	10,506	64,791	19,178	
	Combination	11,654	64,256	13,743	
	Control	27,974	79,988	93,843	96h
	Curcumin	32,889	11,304	86,317	
	Drug	2,364	61,327	20,483	
	Combination	3,455	62,408	10,945	
P7	Control	63,678	96,975	96,609	96h
	Curcumin	48,452	97,387	93,463	
	Drug	14,702	97,954	79,674	
	Combination	16,818	96,441	67,742	
P8	Control	46,378	70,509	97,801	72h
	Curcumin	41,682	66,942	96,602	
	Drug	12,94	67,449	83,632	
	Combination	9,127	57,341	72,459	
P12	Control	33,385	45,241	38,047	72h
	Curcumin	11,495	22,923	75	

	Drug	5,507	41,805	45,083	
	Combination	2,255	32,929	35,787	
P13	Control	57,348	59,767	99,172	72h
	Curcumin	37,935	60,562	96,856	
	Drug	3,329	12,416	64,315	
	Combination	1,762	18,149	68,627	
P15	Control	44,266	77,997	91,864	48h
	Curcumin	30,265	76,302	93,805	
	Drug	33,546	73,229	85,167	
	Combination	17,466	74,16	91,169	
P16	Control	48,667	87,332	97,204	96h
	Curcumin	37,675	84,834	96,177	
	Drug	1,194	39,167	32,367	
	Combination	0,905	35,861	25,551	
P17	Control	44,603	81,727	94,852	96h
	Curcumin	24,977	77,387	86,477	
	Drug	2,039	72,035	47,001	
	Combination	1,353	72,874	26,157	
P18	Control	67,087	94,835	92,785	72h
	Curcumin	63,071	94,312	94,349	
	Drug	17,306	91,597	67,822	
	Combination	17,711	91,724	73,777	
	Control	60,959	95,541	97,979	144h
	Curcumin	58,74	96,439	96,498	
	Drug	0,716	95,652	3,283	
	Combination	1,248	93,137	1,404	
P19	Control	69,397	77,761	98,043	72h
	Curcumin	61,818	73,382	96,04	
	Drug	5,167	51,515	44,949	
	Combination	2,385	55,625	2,697	
P20	Control	33,14	82,391	84,589	96h
	Curcumin	30,985	80,422	94,741	
	Drug	2,744	77,168	68,921	
	Combination	1,926	77,157	58,26	
P21	Control	50,947	92,191	81,563	48h
	Curcumin	51,022	92,62	83,4	
	Drug	35,535	90,207	64,903	
	Combination	23,555	92,459	57,9	
	Control	39,945	91,881	96,28	144h
	Curcumin	35,178	93,212	91,618	
	Drug	4,526	92,798	0,636	
	Combination	4,417	94,251	0,452	
P22	Control	55,947	74,07	51,455	48h
	Curcumin	54,908	83,161	63,089	
	Drug	53,028	85,681	33,718	
	Combination	52,081	86,97	42,761	
	Control	28,076	54,709	92,436	144h
	Curcumin	24,895	77,647	92,296	
	Drug	0,388	66,111	3,361	

	Combination	0,224	62,252	8,511	
P23	Control	58,471	66,842	98,659	72h
	Curcumin	38,418	63,693	95,568	
	Drug	1,677	30,173	30,743	
	Combination	1,167	29,709	12,887	
P25	Control	43,492	92,383	21,826	96h
	Curcumin	20,998	70,605	89,436	
	Drug	2,325	87,308	13,774	
	Combination	2,274	81,276	5,248	
			**IBRUTINIB**		
P1	Control	48,382	1,246	96,154	96h
	Curcumin	30,323	2,356	96	
	Drug	29,156	1,418	88,732	
	Combination	12,372	0,987	60	
P4	Control	68,43	32,484	94,25	72h
	Curcumin	44,955	28,034	88,361	
	Drug	50,573	16,311	72,428	
	Combination	21,699	5,766	26,446	
	Control	54,008	29,178	98,354	96h
	Curcumin	28,572	19,976	88,254	
	Drug	32,717	6,836	68,008	
	Combination	9,837	4,097	0,289	
	Control	35,711	25,736	96,822	96h
	Curcumin	14,903	21,636	79,063	
	Drug	18,516	4,893	80,886	
	Combination	7,089	1,359	27,273	
	Control	51,938	20,011	97,231	96h
	Curcumin	32,889	11,304	86,317	
	Drug	33,587	7,725	64,498	
	Combination	8,983	2,765	10,084	
P9	Control	29,975	28,385	96,563	120h
	Curcumin	12,085	14,43	51,401	
	Drug	6,691	10,731	62,571	
	Combination	1,464	20,256	9,091	
P10	Control	37,289	3,323	92,661	120h
	Curcumin	12,622	2,829	82,857	
	Drug	14,47	1,24	7,692	
	Combination	4,495	1,064	0	
P24	Control	51,483	30,29	98,658	120h
	Curcumin	47,092	13,207	96,985	
	Drug	22,27	4,587	58,621	
	Combination	5,084	5,975	20,69	
			**RITUXIMAB**		
P2	Control	33,975	0,497	100	96h
	Curcumin	16,82	0,158	100	
	Drug	11,649	0,895	0	
	Combination	14,125	0,699	100	
P3	Control	53,805	31,872	91,704	96h
	Curcumin	33,601	22,767	85,802	

	Drug	54,086	31,919	90,422	
	Combination	31,829	21,345	81,922	
P7	Control	63,678	96,975	96,609	96h
	Curcumin	48,452	97,387	93,463	
	Drug	57,613	98,761	94,145	
	Combination	51,634	97,098	65,606	
P11	Control	33,425	43,738	91,773	120h
	Curcumin	13,835	44,055	88,494	
	Drug	34,304	41,898	84,178	
	Combination	10,739	44,776	79,371	
P17	Control	44,603	81,727	94,852	96h
	Curcumin	24,977	77,387	86,477	
	Drug	49,654	81,71	56,342	
	Combination	30,841	80,646	87,836	
**VENETOCLAX**					
P14	Control	27,186	11,503	71,875	120h
	Curcumin	9,711	9,018	73,387	
	Drug	1,582	21,277	1,111	
	Combination	1,243	29,412	1	
